# Correlation of autoantibody profiles with clinical parameters in exfoliative glaucoma patients

**DOI:** 10.1007/s10792-025-03783-0

**Published:** 2025-10-03

**Authors:** Ryan Potter, Marcelo Ayala, Andreas Tilevik

**Affiliations:** 1 Sahlgenska Academy , Gothenburg University, Gothenburg, Sweden; 2 Systems Biology Research Centre, University of Skövde, Skövde, Sweden; 3 Eye Department, Sahlgrenska University Hospital, Mölndal, Gothenburg, Sweden; 4 Karolinska Institutet, Stockholm, Sweden

**Keywords:** Biomarkers, Ophthalmology, Autoimmune, Proteomics, Pseudoexfoliation glaucoma, Systems Biology

## Abstract

**Background:**

Exfoliative glaucoma (XFG) is characterized by elevated intraocular pressure (IOP) caused by fibrous deposits obstructing aqueous humor drainage, traditionally emphasizing mechanical factors. In this study, we aim to investigate the relationships between clinical parameters and autoimmune activity in exfoliative glaucoma (XFG) patients, and identify patterns of autoantibody reactivity among patient subgroups with differing levels of overall autoimmune activity.

**Methods:**

We analyzed serum autoantibody profiles against 92 antigens in 116 XFG patients using an xMAP suspension bead array. Spearman correlation analysis and PERMANOVA were applied to evaluate relationships between autoantibody reactivity and clinical parameters in glaucoma management. Hierarchical clustering was used to identify clusters of patients based on overall autoantibody reactivity levels, and network analysis using shortest path mapping was utilized to explore potential connections among the proteins associated with the antigens used in this study and other proteins not targeted in this study.

**Results:**

Significant positive correlations were found between the clinical parameter SE and autoantibodies targeting the proteins LOXL3, CYP39A1, and HYOU1. Clustering analysis revealed distinct subgroups of patients with differing overall autoantibody reactivity levels, notably showing significant negative correlations between broad autoantibody profiles and CCT exclusively within the subgroup characterized by higher autoimmune activity. Network analysis identified CTNNB1 as a prominent multi-path intermediary connecting disparate proteins, highlighting potential common regulatory pathways.

**Conclusions:**

This study identifies novel correlations between clinical parameters and autoimmune activity in XFG, suggesting refractive changes and corneal structural characteristics may be influenced by specific immune mechanisms. These findings underscore the value of integrating immunological profiling with clinical glaucoma assessments, potentially enhancing patient stratification and identifying possible therapeutic targets.

**Supplementary Information:**

The online version contains supplementary material available at 10.1007/s10792-025-03783-0.

## Introduction

Exfoliative glaucoma (XFG) is a type of secondary open-angle glaucoma characterized by the buildup of fibrous deposits in ocular tissues, especially within the trabecular meshwork (TM) and on the lens surface [1]. These deposits can obstruct the outflow of aqueous humor, causing increased intraocular pressure (IOP), which is widely recognized as a key factor in progressive optic nerve damage seen in XFG patients [2, 3]. While mechanical factors like TM dysfunction have traditionally been central to understanding XFG, recent research highlights the importance of immunological processes, specifically autoantibody production, in potentially driving both disease onset and progression [4]. Moreover, the damage in glaucoma is now understood to be multifactorial; in addition to mechanical stress, oxidative stress, ischemia, and metabolic dysfunction contribute to neuronal injury. For instance, maintaining NAD⁺ homeostasis is essential for protecting retinal ganglion cells, and targeting the enzyme NMNAT2 has been shown to confer neuroprotection by modulating these metabolic pathways [5, 6].

In a previous study, we aimed to identify potential biomarkers for XFG using autoantibody profiling in a case–control design, with 30 XFG patients and 30 healthy controls [7]. In this study, we identified seven antigen fragments which had a significant antibody reactivity in XFG patients (DGCR2, LOX, FUT2, LGSN, ANXA10, TMEM9B). These were able to successfully classify XFG patients from controls with a relatively high accuracy (AUC = 0.85), indicating their potential as indicators of the disease. Several other studies have also demonstrated distinctive autoantibody profiles in XFG patients, suggesting that immune responses may be linked with pathological changes in ocular tissues [8–10]. One prominent hypothesis is that autoantibodies may arise as part of a protective mechanism against misfolded or abnormal proteins. This response could help neutralize potentially harmful aggregates, as seen in various neurodegenerative diseases where similar autoantibodies assist in clearing misfolded proteins [11]. However, autoantibodies can also drive inflammation, activating immune cascades and recruiting inflammatory cells, thereby potentially exacerbating tissue damage [12]. Understanding this balance—whether autoantibodies predominantly protect or harm ocular tissues—is essential for unraveling their role in XFG pathogenesis. Moreover, while low-level autoantibody production is a normal facet of immune surveillance in healthy individuals [13], in glaucoma the sustained cellular stress and tissue injury may not only trigger a broad, non-specific autoimmune response but also promote the generation of more specific autoantibodies [8, 14, 15]

Clinical parameters such as presence of diabetes, hypertension or migraines, age, sex, smoking status, cup-to-disk ratio (CD), optical coherence tomography (OCT), spherical equivalent (SE), central corneal thickness (CCT), visual acuity (VA), and visual field index (VFI), mean deviation (MD), rate of progression (ROP) guided progression analysis (GPA), and whether the patient required selective laser trabeculoplasty (SLT) are routinely measured in glaucoma management and offer valuable insights into disease severity and structural integrity of the eye [16, 17]. Yet, the relationship between these clinical parameters and autoantibody profiles remains unexplored in XFG. Establishing these correlations could identify previously overlooked immunological influences on structural or functional eye changes, offering new perspectives beyond mechanical explanations. Integrating these immunological insights with established clinical measurements could ultimately improve patient stratification and open new avenues for targeted therapeutic interventions.

In this study, we aim to investigate the relationships between clinical parameters and autoimmune activity in exfoliative glaucoma (XFG) patients. Additionally, we seek to identify patterns of autoantibody reactivity among patient subgroups with differing levels of overall autoimmune activity. Furthermore, by employing network-based analytical methods, we investigate potential interactions and shared pathways among seemingly disparate antigens. Through this multifaceted approach, we hope to enhance the understanding of autoimmune mechanisms in XFG and their potential clinical implications.

## Materials and methods

### Patients

In this prospective non-randomized cohort study, we enrolled patients diagnosed with XFG at the Ophthalmology Department of Skaraborg Hospital in Skövde and Sahlgrenska University Hospital in Gothenburg, Sweden. The enrolment period lasted from January 1, 2014, to December 31, 2017. All patients were followed for a duration of three years, with a variation of plus or minus three months. Written informed consent was obtained from each patient, and the study protocol received ethical approval from the University of Gothenburg (Approval Number: DN:119–12 and amendment 2023–03742-02). The research was conducted in accordance with the principles outlined in the Declaration of Helsinki.

During the recruiting visit, an ophthalmic nurse checked patients' visual acuity using a Snellen chart and performed a visual field test. The Humphrey field analysis (Carl Zeiss, Carl-Zeiss-Straße 22, 73,447 Oberkochen, Germany) was conducted with the threshold 24–2 strategy. Following this, an ophthalmologist (MA) measured intraocular pressure (IOP) with a Goldmann applanation tonometer and conducted a slit-lamp biomicroscopy examination, including gonioscopy.

Pupils were dilated with 2.5% phenylephrine and 0.5% tropicamide (Bausch & Lomb UK Ltd., 106 London Road, Kingston-upon-Thames, Surrey KT2 6TN, England). After 20 min, the presence of exfoliation was confirmed, and the optic nerve was assessed using a 90-D lens. Central corneal thickness (CCT) was then measured using an ultrasound device (Tomey Pachymetry; Tomey Corp, Nagoya 451–0051, Japan). At the end of the visit, blood samples were collected. The number of medications was recorded based on the number of compounds rather than the number of bottles used.

All patients at the recruiting visit filled out a questionnaire about family history for glaucoma, hypertension, diabetes, migraine and smoking. The answers were rated as "yes" or "no".

Inclusion criteria:

1. Participants must have a diagnosis of XFG based on the criteria established by the European Glaucoma Society (EGS) (17). This includes:

- An untreated IOP of 21 mmHg or higher.
- An open anterior chamber angle.
- Glaucomatous visual field defects, confirmed by at least two repeatable Humphrey 24–2 tests.
- Glaucomatous optic nerve damage.
- The presence of exfoliation material.

2. Participants must have completed at least five reliable visual field tests during the three-year follow-up. Reliability is defined as having false positives ≤ 15%, false negatives ≤ 20%, and/or fixation losses ≤ 30%.

3. Participants must be aged 85 years or younger at the time of recruitment.

Exclusion criteria:

1. Participants diagnosed with advanced glaucoma, defined as a MD of 18 dB or greater and/or a VFI of 40% or less. This is due to 'floor effects,' which indicate that further losses in the visual field cannot be detected [18, 19].

2. Participants with a history of glaucoma surgery, except for uneventful cataract surgery or SLT.

3. Participants with other eye conditions (e.g., central venous occlusion, retinal detachment, etc.) that could impact visual fields during the three-year follow-up.

4. Participants suffering from autoimmune diseases and/or treated with immunosuppressive medications (self reported).

Visual field progression assessment

The progression of the visual field was studied using three different methods.

The first method involved the mean deviation (MD) values. We calculated the difference in MD values from the beginning (MD Diagnosis) to the end (MD 3) of the study, with lower negative values indicating greater progression. MD values were chosen because several studies have identified them as an essential indicator of progression [20–22].

The second method utilized the visual field index VFI at the time of diagnosis (VFI Diagnosis) and after three-year follow-up (VFI 3). The device calculated the VFI and performed a regression analysis to determine the rate of progression (ROP). The ROP was calculated as the percentage of VFI deterioration per year during the three-year period.

The third method was the guided progression analysis (GPA). This method is also performed automatically by the device (GPA Alert) and is distinct from ROP. While ROP is considered a "trend analysis," GPA is classified as an "event analysis." The device compares each individual point during examinations and provides progression results as 'no', 'possible', or 'likely'.

### Antigen selection for xMAP assay

The selected antigens for the current study were based on our previous study [7] where we initially began with 3072 candidate antigens including those associated with genes expressed in the eye, combined with a random selection from the Human Protein Atlas. Based on the results from the array, 60 antigens were selected which showed reactivity in the samples together with 45 additional antigens selected via literature search.

To identify antigens for the current study, we began by selecting antigens identified as significantly differentially expressed in our previous study with adjusted *p*-values less than 0.05 [7]. The proteins associated with these antigens formed the foundational dataset for subsequent analyses.

In addition to the foundational dataset, we selected genes associated to antigen fragments, in order of priority: genes which were highlighted in other relevant studies, genes corresponding to antigens with significant reactivity before multiple test correction and in the top 50 mean MFI values within the dataset, genes corresponding to proteins which were closely associated to the foundational dataset through network analysis (see below), genes corresponding to antigens which were in the top 50 MFI values and adjusted for the control group, ordered by mean MFI, and genes which were in the top MFI values, ordered by mean MFI. In total, we selected 92 candidate genes which included the seven from our previous study, eight from other studies, 26 which were present in multiple selection criteria, the 10 highest ranked remaining from each criteria list (expression values, p-values, correlation to foundational set), and 21 based on shortest path network analysis between the others.

These candidate genes were further reduced due to antigen availability. In total, 92 antigen fragments were selected that were associated with 67 genes.

### xMAP assay

The xMAP assay was carried out at SciLifeLab, Stockholm, Sweden, using a custom suspension bead array to evaluate the 92 selected antigen fragments. The workflow consisted of antigen coupling, sample preparation, and assay execution, followed by data acquisition and quality control.

#### Antigen coupling to beads

A total of 96 bead IDs were utilized. 92 protein fragments plus 4 controls were immobilized on color coded magnetic beads (MagPlex, Luminex Corp., Austin, TX) using NHS and EDC based chemistry. The antigen fragments were coupled into beads in a 96-well plate. The included controls consisted of His6ABP (control of binding to protein fragment tag, HPA resource), buffer blank (control of binding to bare bead), rabbit anti-human IgG (loading control, 309–005-082, Jackson immunoresearch), and Epstein-Barr nuclear antigen 1 (EBNA1, positive control, high frequency expected, ab138345, abcam). The serum samples were diluted 1:250 in assay buffer (3% BSA and 5% milk in PBS supplemented with 0.05% Tween-20 and 0.16 mg/ml His6ABP tag). The diluted samples were incubated for 1 h at room temperature to pre-block any potential antibodies toward the tag (derived from Streptococcal protein G). Subsequently, the diluted samples were incubated with the antigen bead array for 2 h. The bound antibodies were thereafter fixated using 0.02% paraformaldehyde (43,368-9 M, Alfa Aesar) for 10 min. An R-Phycoerythrin conjugated anti-human IgG (H10104, Invitrogen) was then applied for 30 min to enable a read out using a FLEXMAP 3D (Luminex Corp., Austin, TX). The readout consists of the median fluorescent intensity (MFI) and the number of beads for each antigen (bead ID) in each sample. The MFI is calculated, per sample, as the median of the signals from individual beads with the same bead ID.

### Pre-processing

To ensure high-quality data for analysis, the MFI data underwent a series of pre-processing steps designed to standardize and normalize the dataset while minimizing noise and confounding factors. This workflow was based on methodologies established in our previous work but adapted to integrate patient metadata, which was not present in the previous study [7].

Background noise was calculated based on the empty-well MFI readings across non-control analytes as the mean plus one standard deviation of these wells. This assumed background value was then subtracted from all MFI values in the dataset. Any negative values were then adjusted to zero, and a value of 1 was added to the full dataset to prevent errors in downstream logarithmic transformations.

Patient metadata, provided in a separate file, was integrated with the adjusted MFI data by aligning patient identifiers between the datasets. Due to that sample collection occurred at the start of patient enrollment, our xMAP dataset included all 125 patients initially enrolled in the study and therefore included samples taken from patients that were later excluded from the study. During this step, samples which were included in the MFI data but not the final patient data were excluded from further analysis. In total, nine samples were excluded at this step.

To address technical variability and ensure data comparability, the adjusted dataset underwent multiple normalization steps. Robust Spline Normalization (RSN), implemented using the “lumi” R package (v4.3.2), was applied to adjust the intensity values. A Box-Cox transformation was performed to stabilize variance and correct heteroscedasticity, with negative values offset prior to transformation (23). Following these steps, the final dataset consisted of 92 antigens across 116 patients.

### Data analysis

The workflow for the following analyses was conducted in Python (v3.12.1) and R (v4.1.2). Conda (v24.1.0) was used to maintain the environment for this research; the conda environment file, along with all code used for these analyses, can be found in our github repository (https://github.com/rpotter6298/xmap_2).

Network interaction analysis.

Using protein–protein interaction networks, we analyzed each protein of intertest to uncover connections with other proteins in order to find candidate antigens included in our final list to be analyzed. Unlike traditional analyses that prioritize direct interactions or centrality measures, this approach treats all connection types—regardless of specific biological mechanism—as potentially informative. Connections were derived from a custom Neo4j graph database (v5.24.2) constructed using StringDB data including all protein interactions for homo sapiens. This included diverse relationships such as coexpression, shared pathways, structural analogies, and others.

The rationale for focusing on shortest path networking lies in its potential to highlight nodes reflecting the broader flow of information across the network. While highly recurrent nodes may not directly regulate or mediate communication between nodes of interest, their repeated presence suggests relevance to the overall network topology. This relevance may arise from direct connections or from indirect influences, such as miRNA activity, external factors, or shared regulatory mechanisms. By adopting this generalized approach, we aimed to identify candidates that might otherwise be overlooked in interaction-focused analyses.

### Correlation analysis

#### Clinical parameter correlation analysis

To explore correlations between the amount of autoantibody reactivity to the selected antigens and the clinical parameters, the data underwent a series of normalization and statistical analyses. Two clinical variables were transformed to simplify the interpretation and align with clinical context. VFI Diagnosis and VFI 3 were converted to VFI Loss metrics to ensure that higher values consistently represented worse outcomes. VFI Loss was calculated as 100 minus the VFI value for each patient at diagnosis or follow-up, respectively. Similarly, MD and MD 3 were taken as absolute values, as all original values were negative in our dataset. This transformation allowed for easier comparisons with IOP and ROP, where higher values indicate worse conditions.

Association between autoantibody reactivity and patient clinical parameters was analyzed by Spearman’s rank correlation method for numeric clinical parameter with the python library Scipy (v1.14.1). The p-values were adjusted using the Benjamini–Hochberg method from the statsmodels library (v0.14.2) to account for multiple testing. For categorical clinical parameters, PERMANOVA was used from the scikit-bio library (v0.6.2) to test whether the overall autoantibody profile was associated with differences in categories using 1000-fold permutation testing to evaluate statistical significance. For parameters highlighted as significantly associated, permutation t-testing based on 10,000 permutations was then conducted for each antigen.

#### Patient clustering

A heatmap was constructed using the matplotlib (v3.6.3) and seaborn (v0.13.2) libraries to visualize autoantibody reactivity across the 92 antigens and hierarchical clustering of patients using Euclidean distances with complete linkage was employed using Scipy to identify groups of high and low overall reactivity profiles. Based on these groupings, the same Spearman correlation as previously mentioned was used on each subset of patients to narrow the discovery of correlation patterns within the respective groups. In the low reactivity group, six antigens were removed prior to correlation analysis due to filtering for zero variance in the subset.

#### Protein correlation analysis

In order to analyze the relationship between relative autoantibody profiles, we then scaled the data based on the patients and conducted a Spearman’s rank correlation between the antigens. The correlation coefficients were visualized with a heatmap, and hierarchical clustering using complete linkage was used to identify clusters of antigens.

### Network analysis of protein interactions

Building on the same principles that we used for antigen selection with the same Neo4j graph database, a network-based methodology was also employed to analyze the shortest paths between protein clusters. These shortest path analyses were conducted to uncover indirect relationships among the selected proteins. Connections with strength values greater than 0.15 were included so that even weaker interactions, which may represent indirect or context-specific relationships, were captured. This approach aligns with the study's goal of identifying broader network patterns and uncovering potentially overlooked connections critical to the proteomic landscape. Rather than focusing on direct connections to each node as a starting point, as is common in some approaches such as those used by StringDB, this analysis sought to explore nodes that appeared in multiple unique shortest paths. This methodology was applied to the top 10 most correlated proteins with SE, as well as clusters identified from both the clinical correlations and the protein correlations. Clusters within each group were determined using the “fcluster” method in Scipy, with thresholds manually determined in order to minimize extremely large (> 20) or small (< 3) groupings. These extreme groupings were excluded from shortest path mapping.

## Results

### Patients

At the beginning of the study, 125 patients were recruited. Nine patients were excluded in the follow-up period due to bad collaboration in the visual fields, dementia, did not attend the check-up visits, etc. A total of 116 patients were finally enrolled in this study. The average age of the patients at the time of recruitment was 70.97 ± 6.12 years, with 56 (48%) being men and 60 (52%) women. The average intraocular pressure (IOP) value at the time of glaucoma diagnosis was 32.44 ± 5.58 mmHg. Among the patients, 88 (76%) were phakic, and 28 (24%) were pseudophakic at the time of inclusion. Exfoliation glaucoma was found to be unilateral in 77 (66%) patients and bilateral in 39 (34%) patients. The mean central corneal thickness (CCT) measured 541.40 ± 33.04 µm. At the start of the study, the average mean deviation (MD) was -6.51 ± 4.89 dB, and the average visual field index (VFI) was 85.56% ± 14.40%.

At the three-year follow-up, the average IOP was 16.70 ± 2.83 mmHg. Patients were taking an average of 2.70 ± 0.84 medications. Furthermore, 37 (32%) of the patients had undergone selective laser trabeculoplasty (SLT), compared to 79 (68%) who had not.

In terms of visual field assessment, the GPA indicated that 68 (59%) of patients showed progression, while 48 (41%) showed no progression. The MD was—10.07 ± 6.36 dB, and the mean VFI was 74.92 ± 19.04%. Additionally, the average ROP over the three years was—2.89% ± 2.43% per year.

Binding of autoantibodies in serum samples to 92 antigens were analyzed for all 125 patients recruited to this study. After integrating patient metadata and excluding samples from disqualified patients, 116 patients and 92 antigens remained for downstream analyses. Preprocessing steps included background subtraction, variance filtering, RSN, and a Box-Cox transformation. All subsequent analyses were performed on this curated dataset.

### Correlation analysis

#### Clinical parameter correlation analysis

**Fig. 1:**
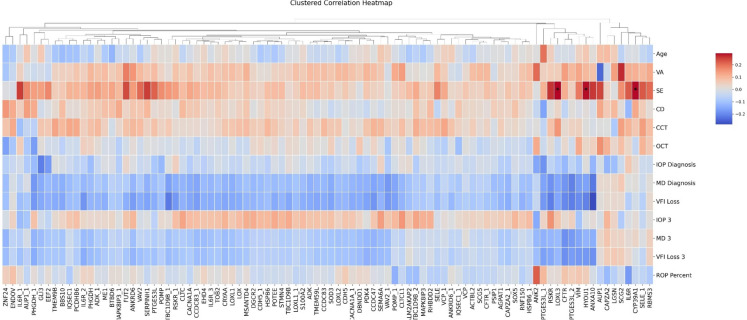
Heatmap showing spearman correlation values between the autoantibodies and clinical parameters. Red color corresponds to positive correlation and blue corresponds to negative correlation values. For proteins with multiple corresponding antigen fragments, subsequent fragments are denoted with an underscore followed by a number. Asterisks represent statistically significant correlation values (adjusted *p* < 0.05) after multiple tests correction using the Benjamini–Hochberg method. High values represent stronger correlation between antigen reactivity and the corresponding clinical parameter

Spearman’s rank correlation was applied to identify associations between the amount of autoantibody reactivity and the numeric clinical variables. VFI and MD measurements were inverted for visual interpretability. The resulting correlation heatmap (Fig. 1) illustrates both the direction and strength of these relationships, with statistically significant (adjusted *p* < 0.05) correlations denoted by asterisks. The only significant correlations were identified between the clinical parameter SE and antigens associated with LOXL3, CYP39A1, and HYOU1. Correlation coefficients for LOXL3, CYP39A1, and HYOU1 were 0.294, 0.290, and 0.289, respectively, and the BH adjusted p-values for all three were 0.047. Thus, the amount of reactive autoantibodies against these three antigens show a significant positive correlation to SE. Although most correlations between individual antigen reactivity and the clinical parameters are insignificant, the overall pattern is consistent for some clinical parameters. For example, the VFI loss and MD are associated with an overall negative correlation to the antigens. Thus, a greater loss to the visual field, as well as a greater absolute MD, is associated with an overall reduction of autoreactive antibodies. However, all correlations are weak since they span in ranges between − 0.3 and 0.3.

To analyze if there was a difference in means between two or more groups in the categorical clinical variables (e.g., sex, diabetes, hypertension) and autoantibody reactivity for all antigens, a PERMANOVA was conducted based on 1000 permutations (Table 1). Out of the seven categorical clinical parameters, only sex showed statistically significant difference in location of the centroids in the multivariate space (adj *p* = 0.019). A permutation t-test based on 1000 permutations was performed on each antigen in relation to sex. However, no significant difference was found across any specific antigen after multiple tests correction.

**Table 1:** PERMANOVA of the categorical clinical parameters and all 92 autoantibodies. Asterisk represents traits with statistically significant differences in centroid location (p <0.05) after 1000 permutation testing.

Parameter	Test statistic	*P*-value
Sex*	3.814	0.019
Diabetes	0.505	0.636
Hypertension	0.591	0.552
Migraine	1.382	0.213
Smoking	0.461	0.836
SLT	0.479	0.681
GPA	2.391	0.055

#### Patient clustering

In order to refine the correlation results to subsets of patients with more similar overall autoantibody reactivity, we applied hierarchical clustering using Euclidean distance with complete linkage across patients based on the MFI values for all antigens. A heatmap was created to visualize these groupings (Fig. S1, supplemental data). This identified three distinct clusters within our cohort, those with relatively high overall autoantibody reactivity (n = 35), relatively low overall autoantibody reactivity (n = 36), and intermediate reactivity (n = 45).

**Fig. 2:**
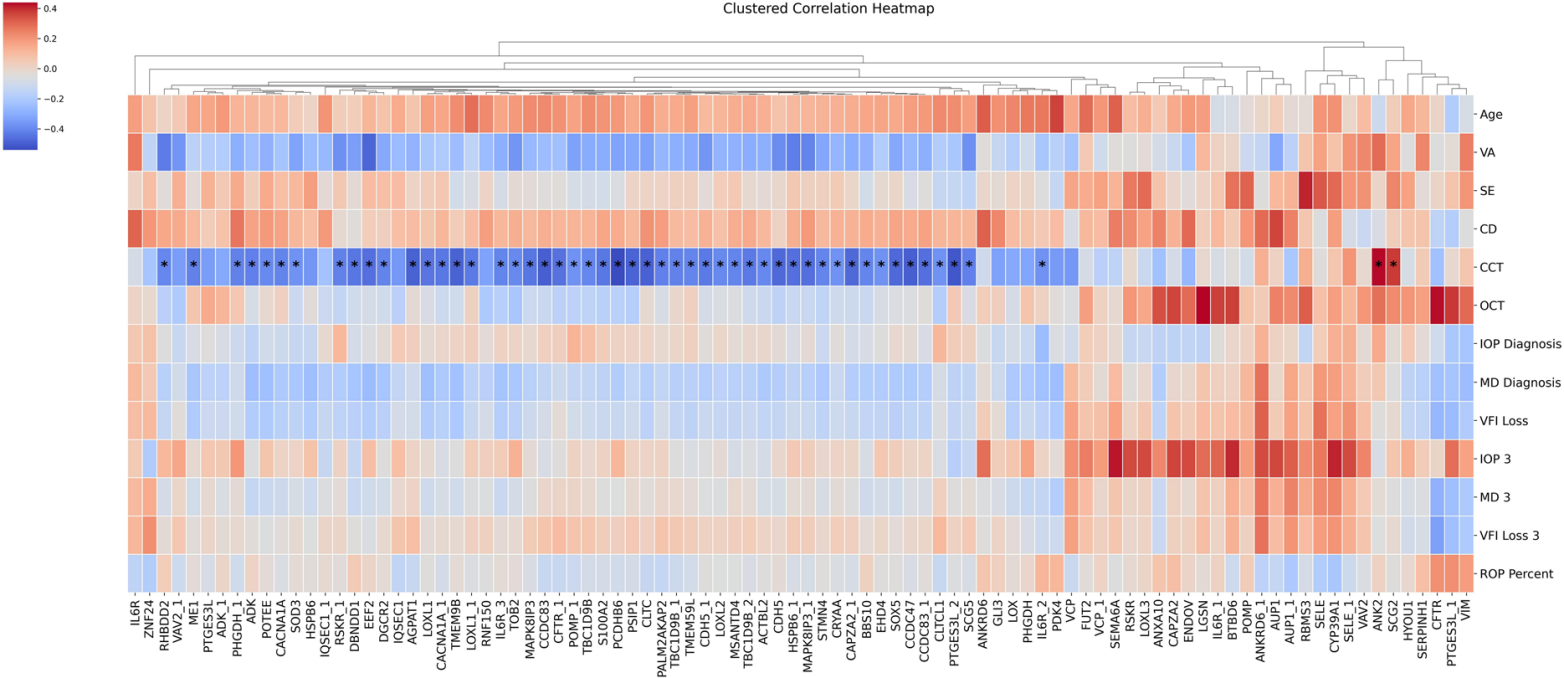
Heatmap showing spearman correlation values between the antigens and the clinical parameters for high reactivity patients. Red color corresponds to positive correlation and blue corresponds to negative correlation values. For proteins with multiple corresponding antigen fragments, subsequent fragments are denoted with an underscore followed by a number. Asterisks represent statistically significant correlation values (adjusted *p* < 0.05) after multiple tests correction using the Benjamini–Hochberg method. High values represent stronger correlation between antigen reactivity and the corresponding clinical parameter

**Fig. 3:**
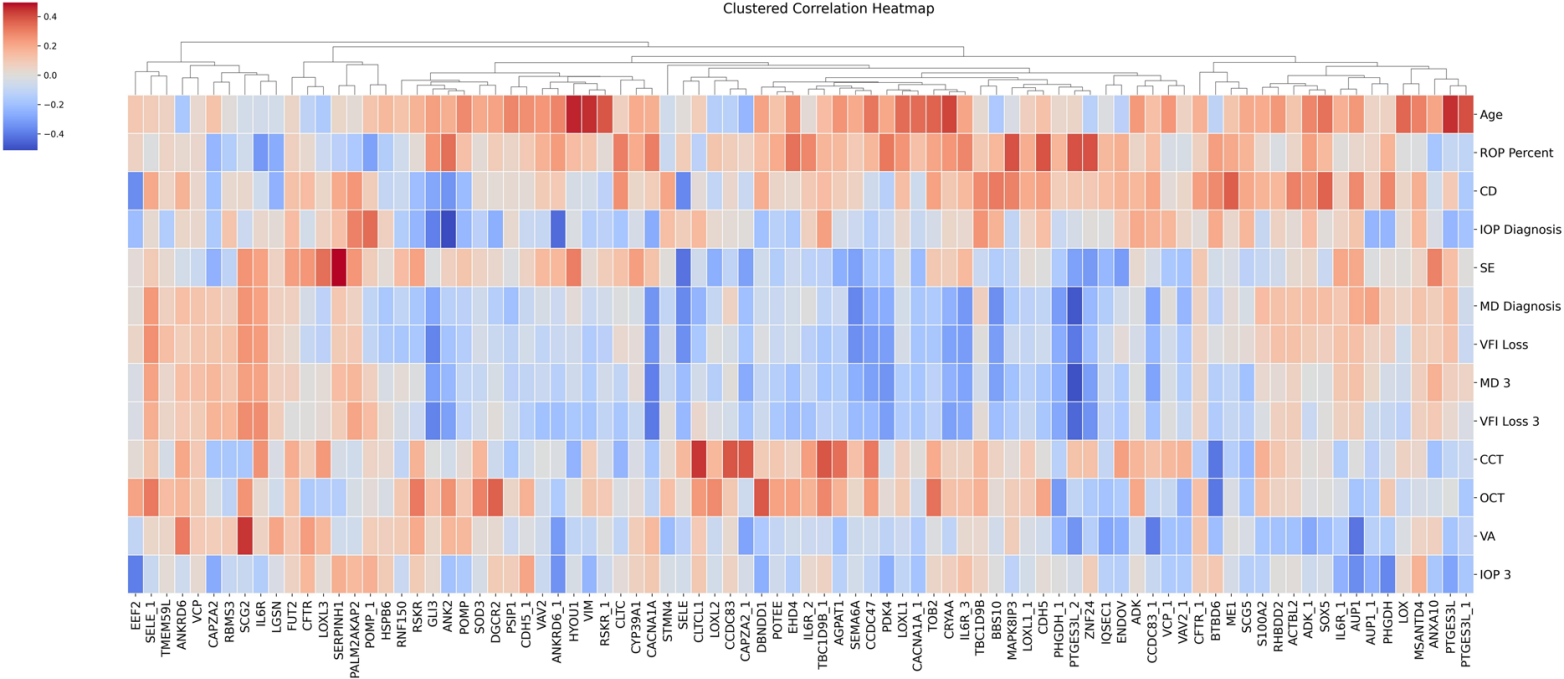
Heatmap showing spearman correlation values between the antigens and clinical parameters for low reactivity patients. Red color corresponds to positive correlation and blue corresponds to negative correlation values. For proteins with multiple corresponding antigen fragments, subsequent fragments are denoted with an underscore followed by a number. High values represent stronger correlation between antigen reactivity and the corresponding clinical parameter

We then applied the same spearman’s rank correlation analysis as previously used on all patients to each of the high and low groups from this clustering analysis. In the high-reactivity group (Fig. 2), consistent negative correlations were found between 46 antigens in one cluster and the clinical parameter CCT. However, the antigens in the other clusters did not show such patterns. In the smallest and most distant of these additional clusters, two antigens (SCG2, ANK2) showed significant positive correlations with CCT. Other apparent patterns also emerge, such as a general negative correlation between several antigens and VA. However, these apparent patterns did not rise to the level of statistical significance following multiple tests correction. In the low reactivity group (Fig. 3), six antigens (IQSEC1, PCDHB6, TMEM9B, TBC1D9B, MAPK8IP3, and HSPB6) were removed due to having no variance within this subset of patients. No clear patterns or significant correlations were identified in the low reactivity group.

#### Protein correlation analysis

**Fig. 4:**
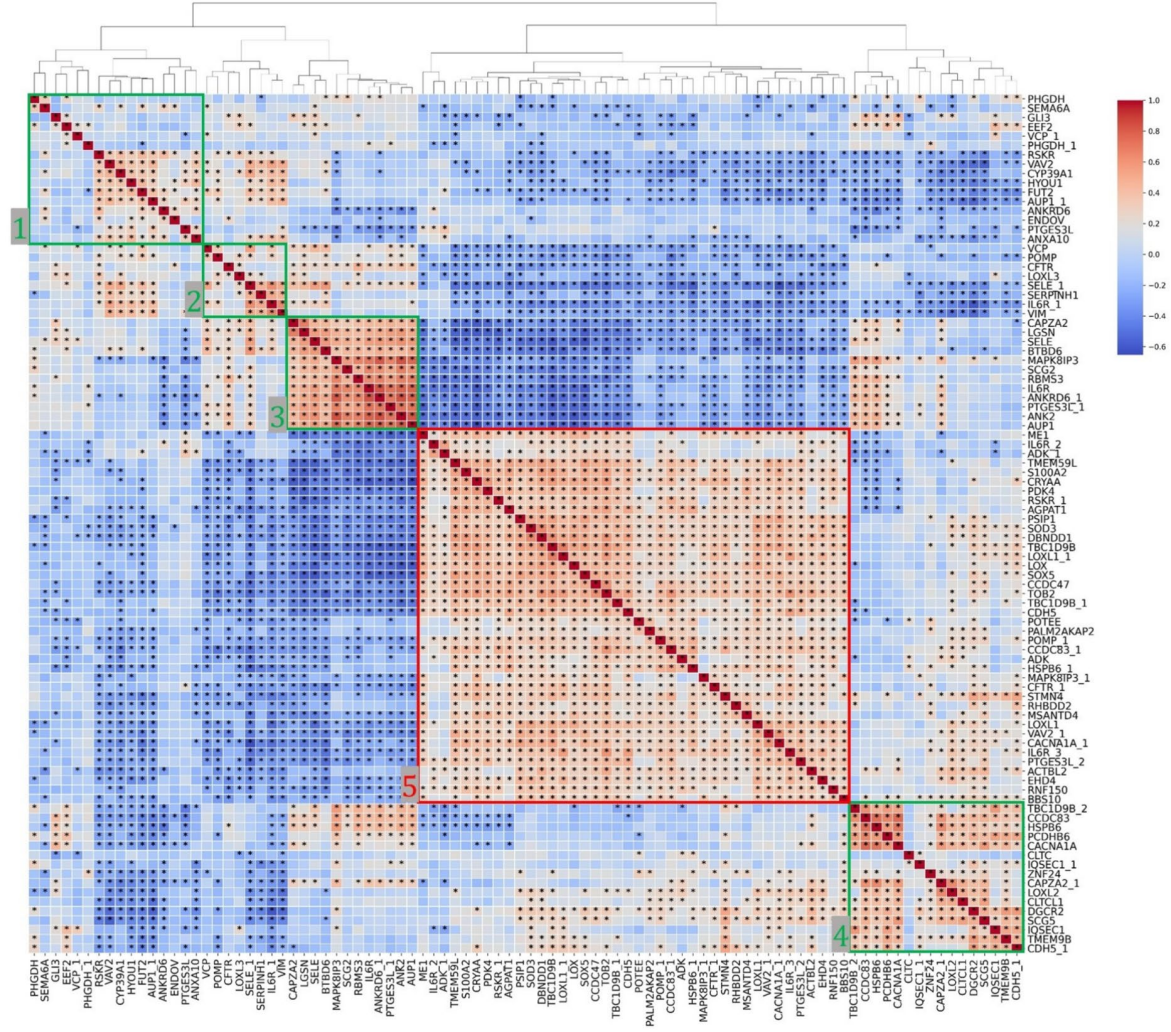
Protein correlation heatmap based on patient-scaled MFI values. Red color corresponds to positive correlation and blue corresponds to negative correlation coefficients. For proteins with multiple corresponding antigen fragments, subsequent fragments are denoted with an underscore followed by a number. Asterisks represent statistically significant correlations (adjusted *p* < 0.05) after multiple correction using the Benjamini–Hochberg method. Green squares represent clusters, based on hierarchical clustering, utilized for shortest path mapping. Red square indicates cluster not used for shortest path mapping

To analyze the relationship between the antigens used in our study to measure the amount of autoantibodies in the patients, Spearman’s rank correlation was computed between pairwise antigens scaled by patient (Fig. 4). This showed that the majority of antigens were significantly associated with other antigens, indicating a relationship between the amount of autoantibodies to the corresponding antigen fragments. The dendrogram identified five clusters of antigen fragments. Clusters 1 and 2 together encompass a visually distinct region which contains all three of the antigens which correlated with SE in the full dataset. Among others, FUT2, a key antigen of interest from our previous study, also appears in this region, and is significantly correlated with the broad majority of members within both cluster 1 and cluster 2. Interestingly, LOX and both antigens associated with LOXL1 clustered with similar correlation profiles in cluster 5, however LOXL2 and LOXL3 differed considerably, appearing in cluster 4 and 2, respectively. The two antigens observed to have positive correlations with CCT in our high-reactivity group (ANK2, SCG2) appear in cluster 3, which appears to be the cluster with the strongest correlation values, on average.

### Network analysis

**Fig. 5:**
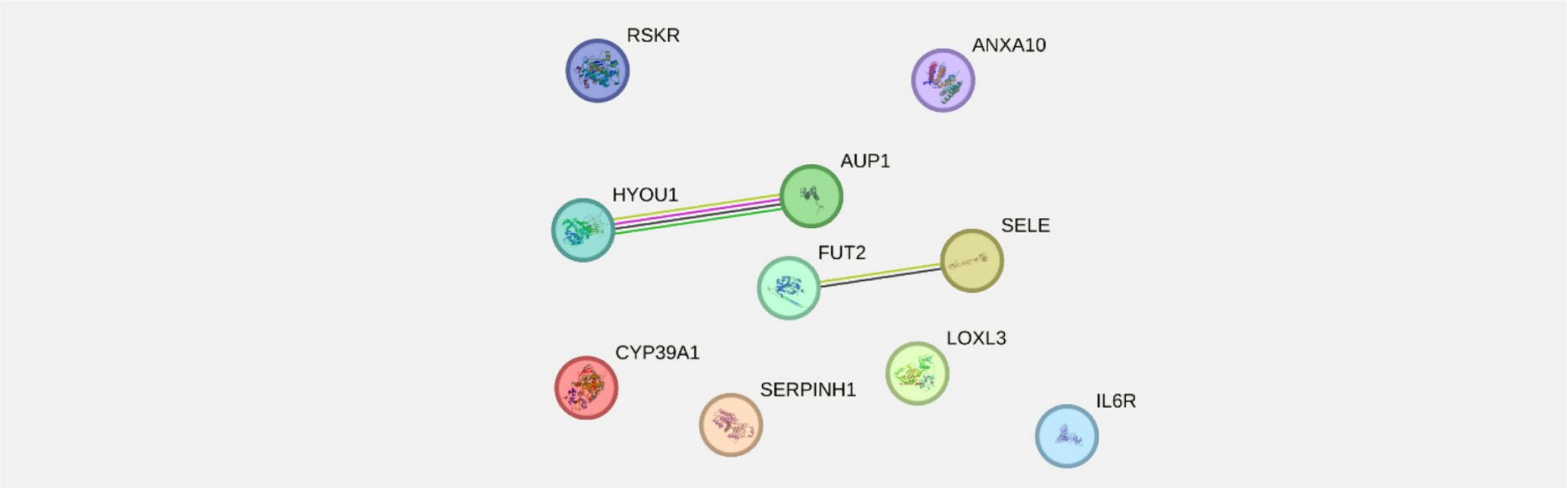
Protein–protein interaction map of top 10 correlating proteins with SE in the full patient cohort based on stringdb web interface using default parameters. Connection colors correspond to interaction source within string’s database

Based on the results from our initial correlation analysis of antibody binding of antigens compared with clinical parameters in the full dataset, we sought to explore potential connections between the top 10 antigens with the strongest positive correlations with SE. We first used String-DB to analyze any direct protein–protein interactions in this group and explore any potential enrichments (Fig. 5). Within this subset, only two direct connections existed, between HYOU1 and AUP1, and between SELE and FUT2.

To identify potential “bridge” nodes in the broader protein–protein interaction network, we performed shortest path mapping using the same methodology we used during the protein selection process. For this visualization, antigen proteins included in this study are represented with triangular nodes, while circles represent proteins involved in the pathing algorithm which were not analyzed in this study. Both the nodes and edges are colored using discrete color scales to indicate the overall frequency of recurrence in the cumulative shortest paths. This analysis highlight CTNNB1 as a highly recurrent node connecting these nodes with the most repeated connections being between CTNNB1 and RBMS3, and between CTNNB1 and CYP39A1.

Using hierarchical clustering with our protein correlation analysis dataset, we identified five clusters of proteins. Four of these were used for shortest path network analysis (Supplementary Data, Figs S2–S5). Cluster 5 was not used for shortest path mapping due to the size of the cluster being too large to provide meaningful results. The shortest path analyses highlighted ACTB in cluster 1, CTNNB1 in cluster 3, and ARF5 in cluster 4 as highly recurrent nodes.

## Discussion

While substantial research has been focused on the differential expression of autoantibodies in XFG [7, 9, 14, 15] the translation of these autoimmune connections to physically observable clinical traits remains underexplored. In this study, we aimed to bridge this gap by analyzing how autoantibody profiles correlate with various clinical measurements across a cohort of 116 XFG patients.

Our findings indicate that certain autoantibody responses in XFG patients are significantly correlated with refractive status, measured as SE (Fig. 1). In particular, the amount of bound autoantibodies against LOXL3, CYP39A1, and HYOU1 showed significant correlations with SE. This suggests that patients’ refractive error (which reflects ocular structural characteristics) may be linked to underlying autoimmune reactivity.

LOXL3 is of special interest given that LOXL1, a related lysyl oxidase, is the well-known genetic risk factor for exfoliation syndrome and XFG, with a single polymorphism accounting for the majority of cases [24]. LOXL3 and other LOX family members contribute to collagen cross-linking and extracellular matrix (ECM) remodeling in the eye [25]. Autoantibody reactivity to LOXL3 in XFG could therefore signify an immune response targeting ECM components, potentially influencing ocular tissue properties such as scleral rigidity or lamina cribrosa integrity that relate to refractive error. Likewise, CYP39A1 is an enzyme involved in cholesterol and oxysterol metabolism, and its relevance to glaucoma is supported by recent genetic evidence: a rare variant in CYP39A1 has been associated with a higher occurrence of XFG and more severe disease progression [26]. The observed correlation between anti-CYP39A1 antibodies and SE may reflect metabolic or structural differences in eyes (e.g. differences in lens or axial length metabolism) that are pertinent to both refractive state and XFG pathology. HYOU1 is an endoplasmic reticulum chaperone induced by hypoxic stress [27]; its correlation with refractive error and presence of autoantibodies could indicate that eyes with certain refractive profiles experience different levels of cellular stress or tissue remodeling, eliciting an immune response. Notably, glaucoma eyes are known to undergo periods of hypoxia due to impaired ocular perfusion or IOP spikes.

In the full dataset, we also observed general trends linking the overall autoantibody repertoire to functional glaucoma outcomes. Notably, patients with higher levels of most autoantibodies tended to have slower rates of VFI loss and less severe mean deviation (MD) deficits. This pattern raises the possibility of a protective effect from certain antibodies in glaucoma patients. Similar neuroprotective effects have been observed in glaucoma patients in several autoantibodies, such as anti-γ-synuclein and anti-GFAP [28, 29].

Our PERMANOVA analysis revealed that the overall distribution of reactivity was significantly different based on sex (Table 1), consistent with previous findings highlighting sex-specific differences in immune response and autoimmune susceptibility across various diseases [30]. Common factors such as diabetes, hypertension, migraine, and smoking were not significantly associated with the relative pattern of the autoimmune profiles in our cohort. These factors have previously been more broadly linked to systemic inflammation and altered autoimmune responses in other contexts [31–34]. It is important to clarify that the lack of association in the PERMANOVA analysis pertains specifically to differences in the composition or pattern of autoantibody reactivity rather than the overall level or abundance of autoantibodies. Thus, in our study, any potential influence from these inflammatory or autoimmune-linked clinical factors might either be generalized, affecting all assessed autoantibodies uniformly or randomly, or might simply not impact the specific subset of autoantibodies analyzed. Further studies exploring broader autoantibody panels or additional patient subsets could clarify whether such clinical conditions influence autoimmune responses in glaucoma differently or more subtly.

Unsupervised clustering of the patient data provided additional insight into the heterogeneity of autoimmune responses in XFG. We identified at least two discernible patient clusters: a “high-reactivity” cluster characterized by generally elevated autoantibody reactivities across many antigens (Fig. 2), and a “low-reactivity” cluster with overall lower reactivity levels (Fig. 3). These clusters were associated with different clinical correlation patterns. It should be noted that our classification of ‘high’ versus ‘low’ reactivity reflects relative responses within the curated antigen panel and does not necessarily imply globally elevated IgG levels or systemic autoimmunity. In the high-reactivity group, a broad suite of autoantibodies demonstrated a negative correlation with CCT. Patients in this cluster who had higher antibody levels to a majority of the antigens tended to have thinner corneas. Conversely, autoantibodies against two particular antigens, ANK2 and SCG2, showed an opposite relationship to CCT (higher levels of anti-ANK2 or anti-SCG2 were associated with thicker corneas). This is particularly interesting in the context of glaucoma outcomes, as thinner corneas have been extensively linked with the severity of damage from glaucoma [35, 36]. It has been shown that patients with more advanced damage are more likely to have a thin CCT [37] and that thicker corneas may mitigate some degree of risk posed by elevated IOP [38].

This divergent pattern observed here between high- and low- reactivity groups suggests that these particular antibodies might play a regulatory or compensatory role, potentially mitigating some of the tissue damage observed with the broader autoantibody profile. In contrast to the high-reactivity group, the low-reactivity cluster did not show these pronounced correlations, with no apparent relationship to CCT across any antibodies. Given that this relationship seems to only be present in cases of high autoimmunity, these results may support that the autoantibodies are not directly responsible for tissue damage, and in cases where overall autoimmunity is lower, they may act protectively by clearing misfolded or malfunctioning proteins. This aligns with the model for protective autoimmunity outlined by Schwartz & Raposo (2014), which showed that CNS-specific immune cells support the healthy function of neurogenesis in the CNS, while noting that severe inflammation impairs neurogenesis [39]. Furthermore, it has been shown that certain alleles of MHC class II molecules may transport certain misfolded proteins from the endoplasmic reticulum (ER) to the cell surface, stimulating antigen-specific B cells [40]. This process is shown to be associated with autoimmune disease susceptibility [41, 42].While normally, MHC class II molecules are present mainly on antigen-presenting cells, their expression can be induced in epithelial cells by certain cytokines like IFN-γ [43, 44]. Given that misfolded or damaged proteins have been suggested to constitute upwards of 30% of newly synthesized proteins [45, 46], this suggests that in some cases, broad autoimmune responses may be triggered by inflammation in the corneal tissues. Under these conditions, autoantibody production at lower levels may effectively manage the presentation of misfolded proteins without causing substantial inflammation or tissue damage. However, once autoimmune activity surpasses a certain threshold, it could initiate a detrimental feedback loop, exacerbating inflammation and potentially contributing to further corneal thinning and tissue damage.

In this context, the observed significant relationship of ANK2 and SCG2 antibodies with CCT in the high-reactivity cluster is particularly intriguing. SCG2 expression is known to respond to elevated IOP and is proposed to increase vascular permeability, potentially acting as a regulatory mechanism to reduce ocular pressure [47]. Thus, the presence of autoantibodies against SCG2 could reduce this protein's activity, potentially lowering vascular permeability. Although initially counterintuitive, decreased vascular permeability in highly inflamed tissues might slow the infiltration of inflammatory cells or leakage of inflammatory mediators, thereby moderating overall inflammation and tissue damage.

ANK2 downregulation has been reported in pterygium, another eye condition characterized by abnormal tissue growth [48]. Interestingly, while no studies have yet directly linked ANK2 with glaucoma, our previous study identified miRNA 3167 and miRNA 876-5P targets as significantly enriched in glaucoma patients, with ANK2 emerging as a shared target [7].

The absence of a significant correlation between autoantibody profiles and CCT in the low-reactivity cluster reinforces the notion that autoantibodies per se are not inherently detrimental. Rather, their functional impact may depend heavily on the overall context and intensity of the autoimmune response.

Future research should aim to validate these potential regulatory mechanisms through targeted functional studies of SCG2 and ANK2 and associated miRNAs. Additionally, longitudinal studies assessing changes in corneal thickness and autoimmune activity over time would help clarify whether these observed relationships represent protective adaptations or reflect broader pathogenic feedback loops in exfoliative glaucoma.

### Network analysis and shortest path mapping

To expand upon the observed antigens with the strongest correlations with SE, we explored network relationships between these antigen proteins. The initial protein–protein interaction analysis using String-DB identified minimal direct interactions among these proteins, only between the pairs HYOU1:AUP1 and SELE:FUT2 (Fig. 5). The limited direct connectivity suggested that these proteins might not interact directly but rather share common signaling pathways or regulatory mechanisms.

**Fig. 6:**
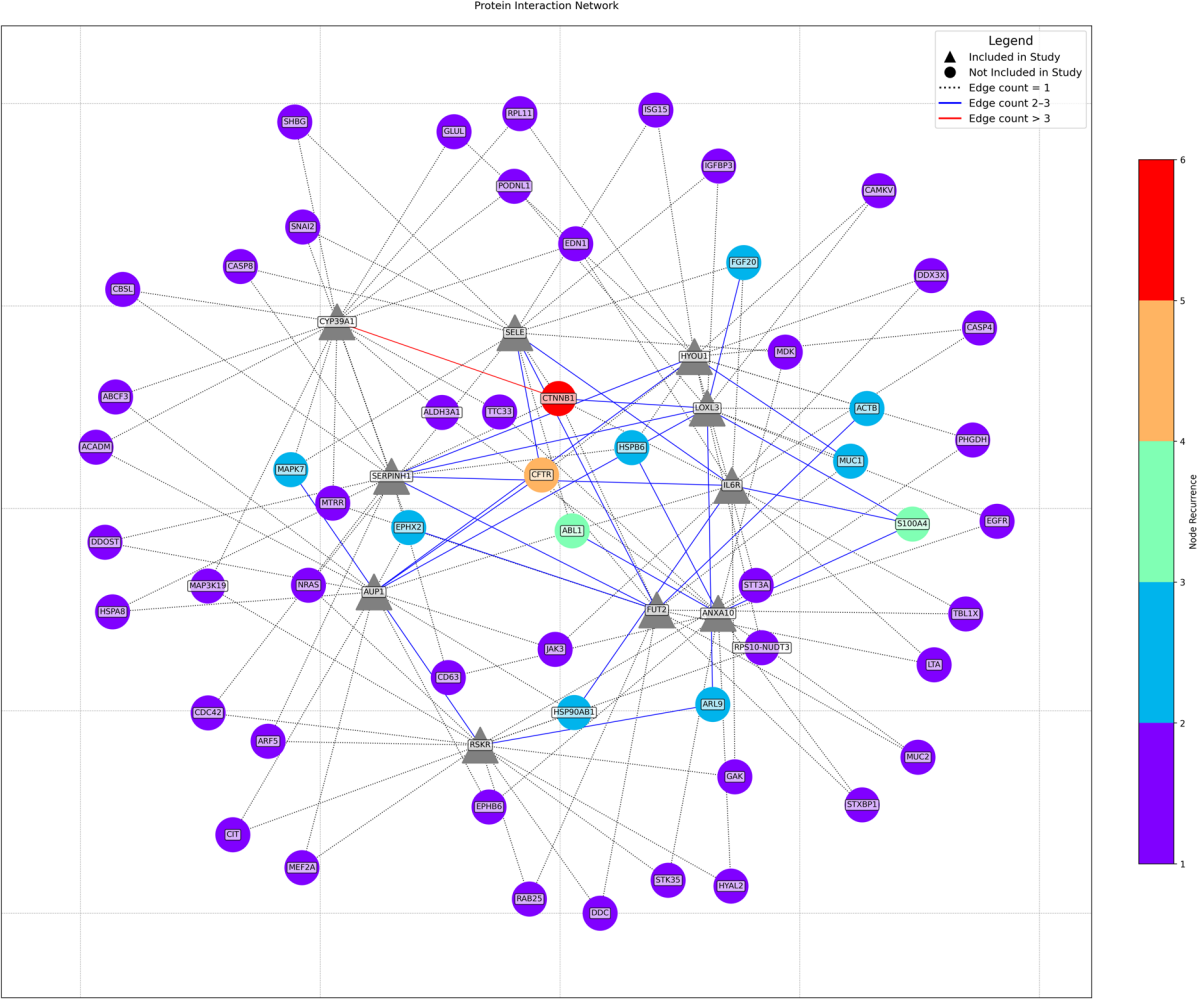
Shortest path mapping using the antigens with the strongest correlations with SE. Node and edge coloring represents the number of shortest pathways that include the node or edge, respectively

Given the sparse direct interactions identified by String-DB, we employed shortest path mapping to uncover potential mediator proteins that might serve as conduits for information flow between indirectly connected proteins (Fig. 6). This analysis prominently highlighted CTNNB1 as a key mediator, frequently serving as an intermediary connecting proteins correlated with SE. While CTNNB1's centrality may partly reflect its broad connectivity within biological networks, its repeated identification as a mediator in our analysis suggests a more specific bridging function. This potential mediator role implies that CTNNB1 could facilitate interactions or coordinate signaling among proteins involved in distinct yet related pathways, contributing to glaucoma pathology.

Notably, the most frequent edges connecting through CTNNB1 involved RBMS3 and CYP39A1. RBMS3 was not highlighted significantly in previous analyses, whereas CYP39A1 exhibited significant correlation with SE, indicating its potential direct relevance in glaucoma. The connection between CTNNB1 and CYP39A1 is particularly intriguing given CTNNB1’s known role in the canonical Wnt signaling pathway—a pathway extensively linked to glaucoma through its regulation of cellular proliferation, differentiation, and survival processes [49–52]. Additionally, CYP39A1 is involved in cholesterol metabolism, at least partially explaining its link to CTNNB1, as cholesterol has been shown to selectively activate canonical, over non-canonical, Wnt signaling [53].

Recent evidence also suggests an intriguing link between Wnt signaling, mediated by CTNNB1, and NAD⁺ metabolism [54]. Targeting the NAD⁺ salvage pathway through enzymes like NAMPT affects β-catenin stability, further highlighting the interaction between Wnt signaling and NAD⁺ metabolism [55]. Given that NAD⁺ levels play a critical role in neuronal protection, as shown by studies involving NMNAT2—a neuronal NAD-producing enzyme whose overexpression confers neuroprotection to retinal ganglion cells [5, 6]—it is plausible that CTNNB1-mediated Wnt signaling might indirectly influence neuronal resilience in glaucomatous conditions through modulation of NAD⁺ pathways. Exploring this connection could yield novel therapeutic strategies for neuroprotection in exfoliative glaucoma.

By clustering antigens that had a similar reactivity to the autoantibodies across the patients (Fig. 4), we identify an interesting overlap between the antibodies correlated with SE in the full dataset and those positively correlated with CCT in the high-reactivity set through the emergence of CTNNB1 as an highly recurrent node in both the SE-correlated group, which is predominantly representative of clusters 1 and 2, as well as in cluster 3, which is a highly correlated group containing ANK2 and SCG2. CTNNB1’s repeated emergence across diverse analyses underscores its likely central role in modulating cellular responses associated with glaucoma.

In the SE-correlated group, we also see FUT2, which was identified in our previous study as a strong classifier for XFG patients [7], significantly correlated with most members of cluster 1 and 2. Recently, FUT2 was shown to fucosylate Wnt2- a process thought to be a key activator of canonical Wnt/β-catenin signaling [56]. Future research should explore potential interactions or shared regulatory pathways involving FUT2 and CTNNB1. Understanding the role of FUT2 in modulating Wnt signaling could significantly deepen our insights into their collective impact on XFG pathology.

### Limitations and considerations

While this study provides novel insights into autoimmune activity in XFG, several limitations should be considered that reflect both the nature of the study design and the complexity of interpreting multi-modal analyses.

From a design perspective, although certain clinical features such as IOP and VFI loss were tracked over time, the primary autoantibody data were collected at a single time point. This limits the ability to assess temporal dynamics or determine causal relationships between immune activity and disease progression. Future longitudinal studies will be required to assess whether the immune patterns identified here precede or follow clinical changes. In addition, the analysis was restricted to XFG patients, without inclusion of a control group. As a result, we cannot assess whether the observed immune patterns are specific to XFG or reflect broader processes such as aging, systemic inflammation, or other ocular conditions.

The analyte panel itself was deliberately curated using prior correlation and network-based selection methods, allowing the study to focus on potentially meaningful targets. However, this targeted approach necessarily excludes a wider array of antigens that might reveal unexpected or novel immune responses in XFG. This narrowing of scope could obscure broader immunological patterns—for example, if inflammatory processes were to induce widespread autoantibody production against a diverse set of tissue-specific proteins, our panel might fail to capture the full extent of that reactivity. As a result, interpretations drawn from clustering or correlation patterns must be understood as partial views of a potentially more complex immunological landscape.

This study employed several analytical approaches, including correlation analysis, PERMANOVA, hierarchical clustering, and shortest-path network mapping. Each of these methods addresses a different aspect of the data and operates under different assumptions. These methods each provide complementary but fundamentally different insights. PERMANOVA evaluates whether the overall pattern or shape of autoantibody expression differs across categorical variables, without addressing absolute levels of antibody production. Correlation analysis, in contrast, pinpoints individual proteins whose expression varies in proportion to clinical measures, offering more specific but narrower associations. Network-based analyses, like shortest-path mapping, operate in a different conceptual space altogether, identifying potential connective nodes within known interaction networks—highlighting proteins of potential interest, but without implying biological causality. In addition, because our network analysis included weaker interaction strengths, a wider range of potentially relevant pathways are captured, though this necessarily reduces the likelihood of direct experimental validation. Together, these methods help triangulate relationships in the data, but their findings are not necessarily expected to overlap or reinforce one another and generally should be regarded as exploratory. For instance, clustering analysis suggests widespread autoimmune reactivity linked to inflammation, yet only a small number of autoantibodies showed significant correlation with clinical variables. This disparity raises the possibility that each analytical approach may be capturing different aspects of the disease. Rather than collectively pointing to a single biological process, these results may reflect multiple, unrelated or only partially connected immune phenomena operating in parallel.

Finally, although the study highlights several intriguing proteins and pathways, such as CTNNB1 and the Wnt/NAD⁺ signaling axis, the proposed mechanistic interpretations remain speculative. Experimental validation, including functional studies in relevant models, will be required to determine whether these pathways play a causal role in disease pathogenesis.

### Conclusions and future directions

This study highlights notable connections between autoantibody reactivity and clinical characteristics in XFG, particularly focusing on the unexpected correlation between SE and specific autoantibodies. Given the ease with which SE can be measured in clinical settings, investigating the basis of this relationship could enable more effective utilization of refractive data for patient assessment and disease monitoring. Additionally, the association between SE-related ocular morphological changes and proteins involved in Wnt signaling pathways presents a promising avenue for future research. Understanding whether and how these structural alterations communicate with immune processes via pathways like Wnt signaling may shed light on potential mechanisms that bridge refractive changes with autoimmune activity.

The clustering analysis further indicates that high levels of inflammation could significantly influence autoimmune responses, potentially leading to generalized or secondary autoantibody production rather than specific, targeted reactions. Recognizing whether immune signatures in highly inflamed conditions reflect primary pathology or secondary inflammation-driven phenomena will be essential for accurately interpreting autoimmune activity and its role in disease progression.

Together, these findings provide a foundation for future research into the interplay between ocular structure, immune signaling, and disease progression in XFG.

## Supplementary Information

Below is the link to the electronic supplementary material.

Supplementary file1 (DOCX 10463 kb)

Supplementary file2 (XLSX 14 kb)

Supplementary file3 (XLSX 15 kb)

## Data Availability

The datasets supporting the conclusions of this article are available in the Github repository, https://github.com/rpotter6298/xmap_2.

## Abbreviations

**CCT**: Central Corneal Thickness
**CD**: Cup-to-Disk ratio
**CNS**: Central Nervous System
**ECM**: Extracellular Matrix
**ER**: Endoplasmic Reticulum
**GPA**: Glaucoma Progression Analysis
**IOP**: Intraocular Pressure
**IFN-****γ**: Interferon-gamma
**MD**: Mean Deviation
**MHC**: Major Histocompatibility Complex
**MFI**: Median Fluorescent Intensity
**OCT**: Optical Coherence Tomography
**PERMANOVA**: Permutational Multivariate Analysis of Variance
**RSN**: Robust Spline Normalization
**SE**: Spherical Equivalent
**SLT**: Selective Laser Trabeculoplasty
**TM**: Trabecular Meshwork
**VA**: Visual Acuity
**VFI**: Visual Field Index
**XFG**: Exfoliative Glaucoma
