# Supplementary material for: Correlation of autoantibody profiles with clinical parameters in exfoliative glaucoma patients
Source: Int Ophthalmol. 2025 Oct 3;45(1):404. doi: 10.1007/s10792-025-03783-0 (PMC12494623; doi:10.1007/s10792-025-03783-0)
Supplement: Supplementary file 1 — Supplementary file1 (DOCX 10463 kb) [file 10792_2025_3783_MOESM1_ESM.docx]

# Supplementary Data

####
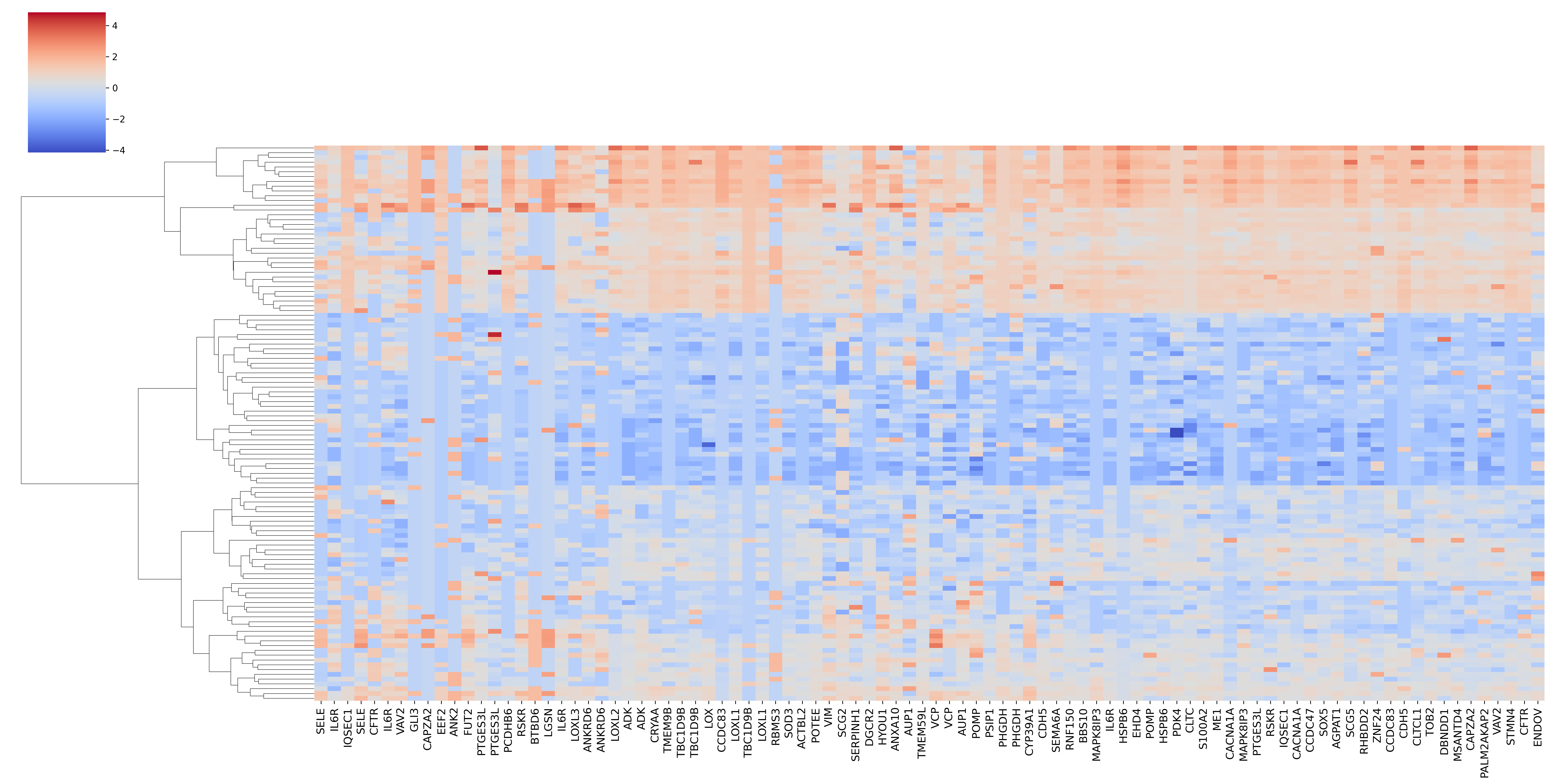


Figure S1 - Heatmap showing normalized MFI values of measured autoantibodies across all patients. Red color corresponds to high MFI values and blue corresponds to low MFI values. Hierarchical clustering was performed using Euclidean distances with complete linkage function to group patients based on reactivity patterns.


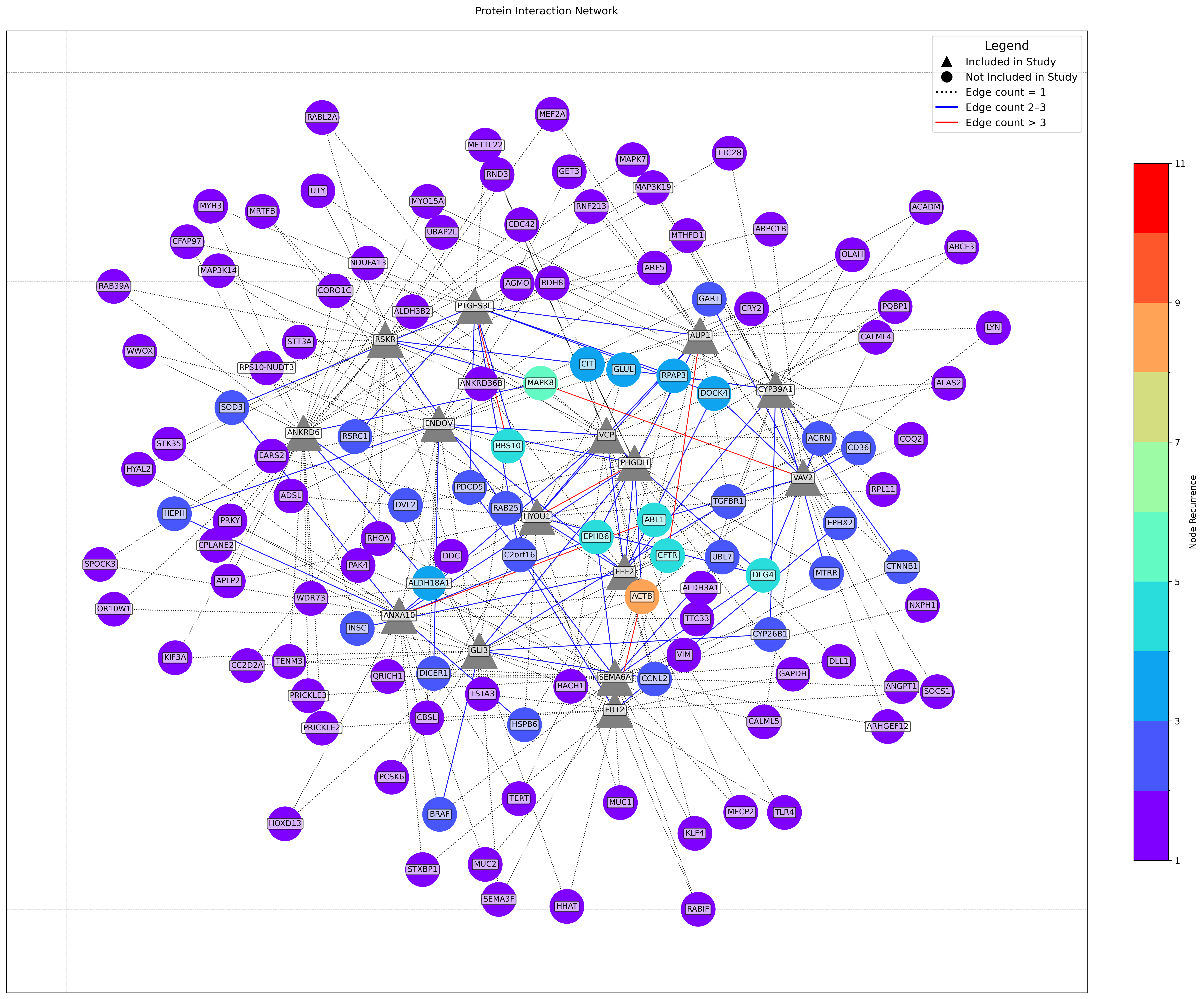


Figure S2 - Shortest path mapping using proteins from protein cluster 1 as starting nodes. Node and edge coloring represents the number of shortest pathways that include the node or edge, respectively.


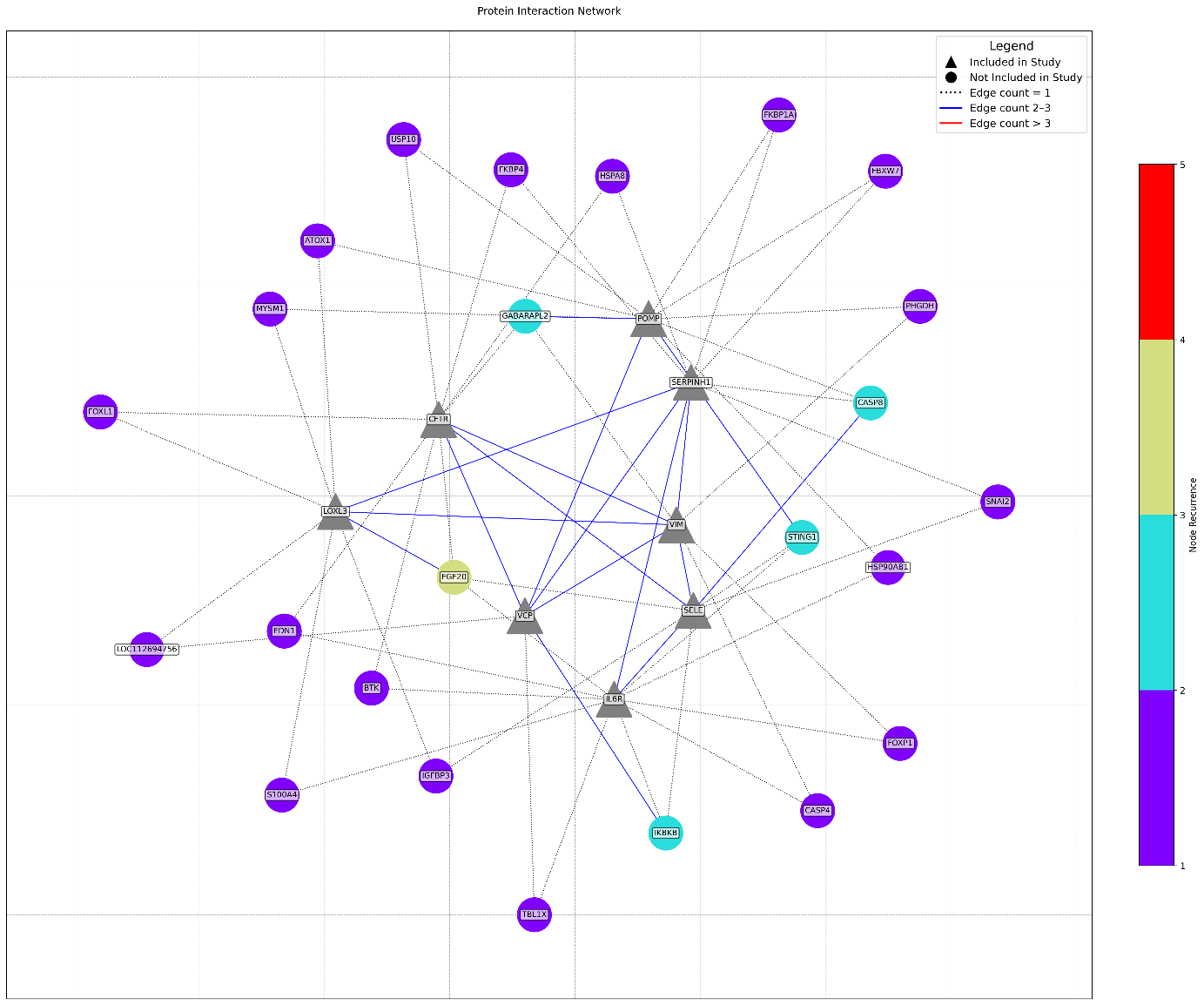


Figure S3 - Shortest path mapping using proteins from protein cluster 2 as starting nodes. Node and edge coloring represents the number of shortest pathways that include the node or edge, respectively.


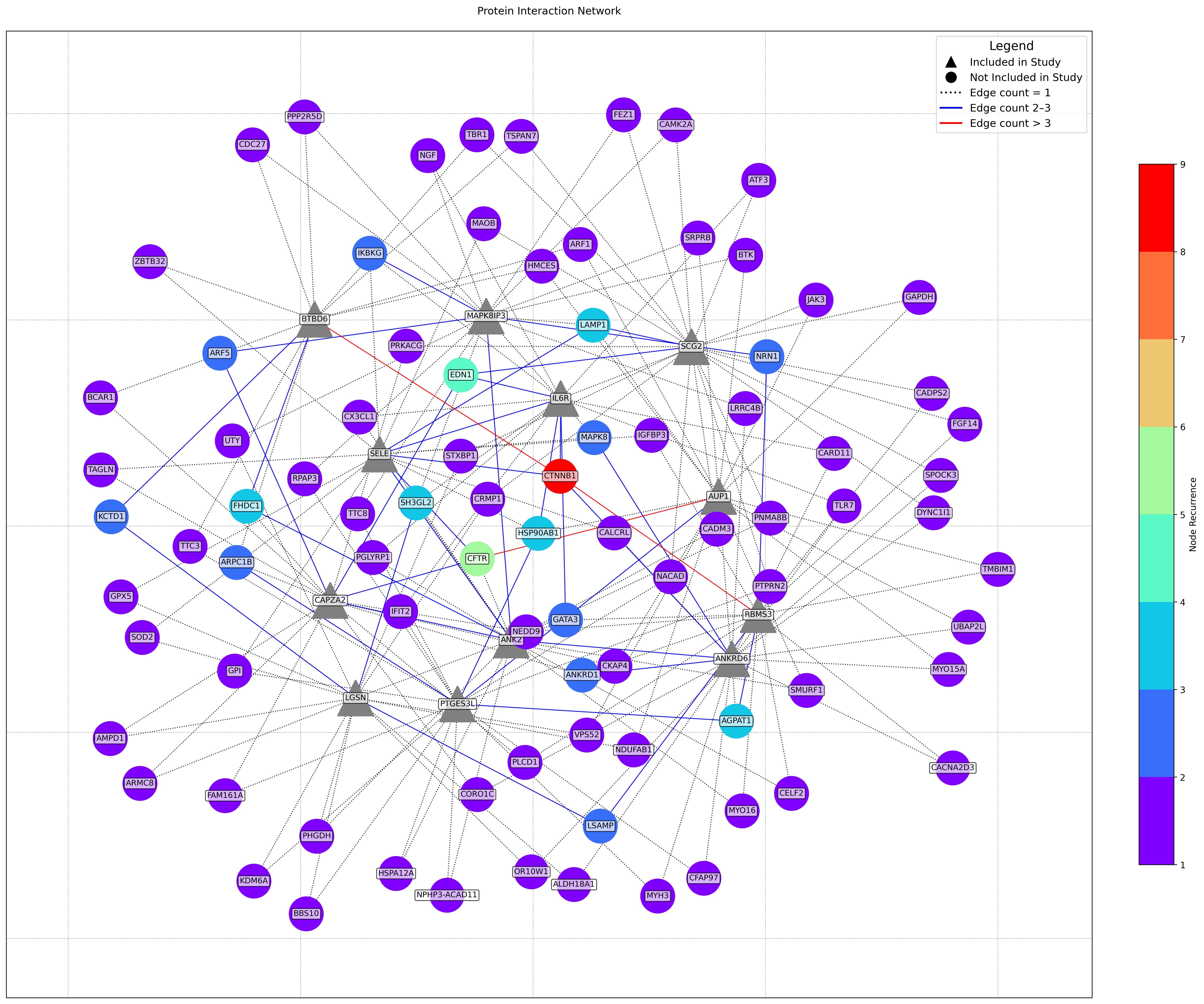


Figure S4 - Shortest path mapping using proteins from protein cluster 3 as starting nodes. Node and edge coloring represents the number of shortest pathways that include the node or edge, respectively.


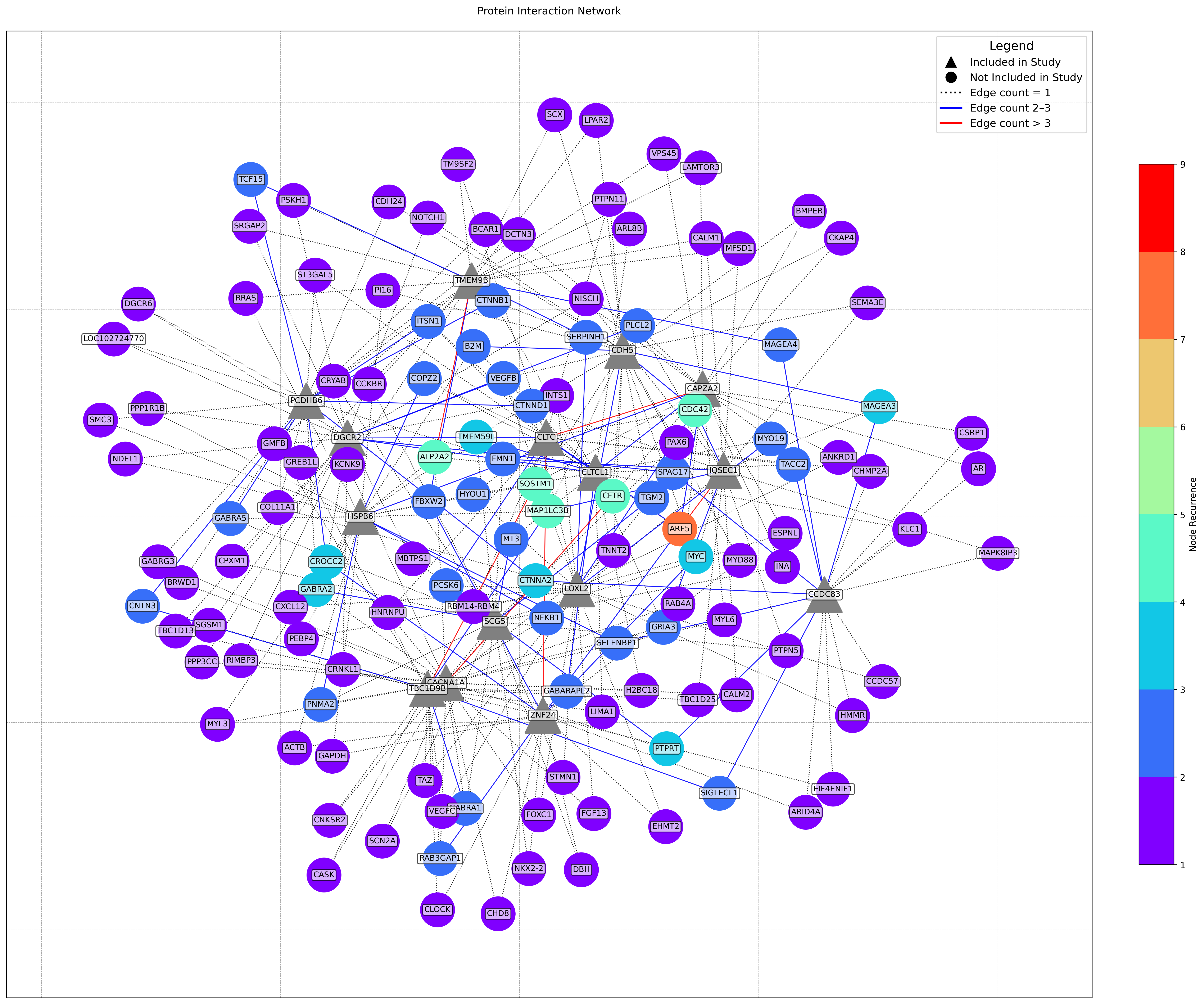


Figure S5 - Shortest path mapping using proteins from protein cluster 4 as starting nodes. Node and edge coloring represents the number of shortest pathways that include the node or edge, respectively.
